# Case Report: Topical imiquimod (Aldara®) as a palliative option for extensive canine facial cutaneous melanocytoma

**DOI:** 10.3389/fvets.2026.1917945

**Published:** 2026-09-11

**Authors:** Hayoung Ryu, Minjeong Kang, Kyu-Woan Cho, Dong-In Jung, Hyeona Bae

**Affiliations:** College of Veterinary Medicine, Gyeongsang National University, Jinju, Republic of Korea

**Keywords:** case report, imiquimod, immunotherapy, melanocytoma, neoplasms

## Abstract

Cutaneous melanocytic neoplasms in dogs generally present as benign lesions, unlike their oral or digital counterparts, but treatment can be challenging when complete surgical excision is restricted by anatomical location. A 5-year-old neutered male Shiba Inu was evaluated for a firm, well-circumscribed, black-pigmented nodule measuring 4 × 3.8 cm on the left facial region, with surrounding pigmented papules. Histopathological examination of punch biopsy specimens confirmed cutaneous melanocytoma with superficial subcutaneous involvement observed in two samples. Complete surgical excision was not feasible due to the broad facial distribution of the nodule and surrounding papules. Consequently, topical immunotherapy with 5% imiquimod cream (Aldara®; 3 M Pharmaceuticals, St Paul, MN, USA) was initiated as a palliative treatment. After 5 months of treatment, the nodule decreased in size to 3.3 × 3.0 cm, became subjectively softer on palpation, and the number of surrounding papules decreased, with no observed adverse effects. Following discontinuation of treatment during a planned washout period before follow-up biopsy, the nodule increased in size, and histopathological findings remained consistent with the initial diagnosis. This case suggests that topical imiquimod may provide temporary clinical improvement and may be considered as a palliative or maintenance option for canine cutaneous melanocytoma when complete surgical excision is challenging.

## 1 Introduction

Melanocytic neoplasms are tumors originating from melanocytes and represent less than 3% of all canine cutaneous tumors (1). Their biological behavior varies according to anatomical location (2). Cutaneous melanocytic neoplasms in dogs are typically benign, in contrast to oral or digital melanocytic tumors, which frequently demonstrate aggressive clinical behavior (3, 4). Surgical resection is generally recommended for cutaneous melanocytic neoplasms when complete removal is feasible (5). For malignant melanocytic tumors, other treatment options, such as radiotherapy, chemotherapy, immunotherapy, and electrochemotherapy, may also be considered (3, 6).

Imiquimod, a nucleoside analogue of the imidazoquinoline family, acts as a toll-like receptor (TLR) 7/8 agonist that enhances innate and cell-mediated immunity responses (7). In human medicine, imiquimod is widely used for several cutaneous neoplastic or pre-neoplastic conditions, and in veterinary medicine it has been reported for equine sarcoids, papillomaviral lesions, feline multicentric squamous cell carcinoma *in situ*, and equine aural plaques (8–14).

Here, we report a dog with an extensive facial cutaneous melanocytoma that presented as a firm, raised, pigmented nodule with multiple surrounding pigmented papules extending from the periocular to auricular region. Because complete surgical excision was considered impractical due to the broad facial distribution of the lesion, topical 5% imiquimod cream (Aldara®; 3M Pharmaceuticals, St Paul, MN, USA) was used as a palliative treatment.

## 2 Case description

### 2.1 Clinical presentation and physical examination

A 5-year-old neutered male Shiba Inu was presented with a firm, pigmented lesion in the left facial region, first noticed by the owner 1 week prior to evaluation. A similar lesion had developed at the same site 18 months earlier and was surgically excised at a referral clinic, with histopathological examination confirming a cutaneous melanocytoma. The histopathology report from the referral clinic described a dense proliferation of melanocytes between the hair follicles and sebaceous glands within the dermis, but did not explicitly state whether the lesion extended into the subcutis. Given the history of a melanocytic neoplasm at the same site, recurrence was considered the primary differential diagnosis, while other cutaneous neoplasms or inflammatory lesions were also considered.

**Figure 1:**
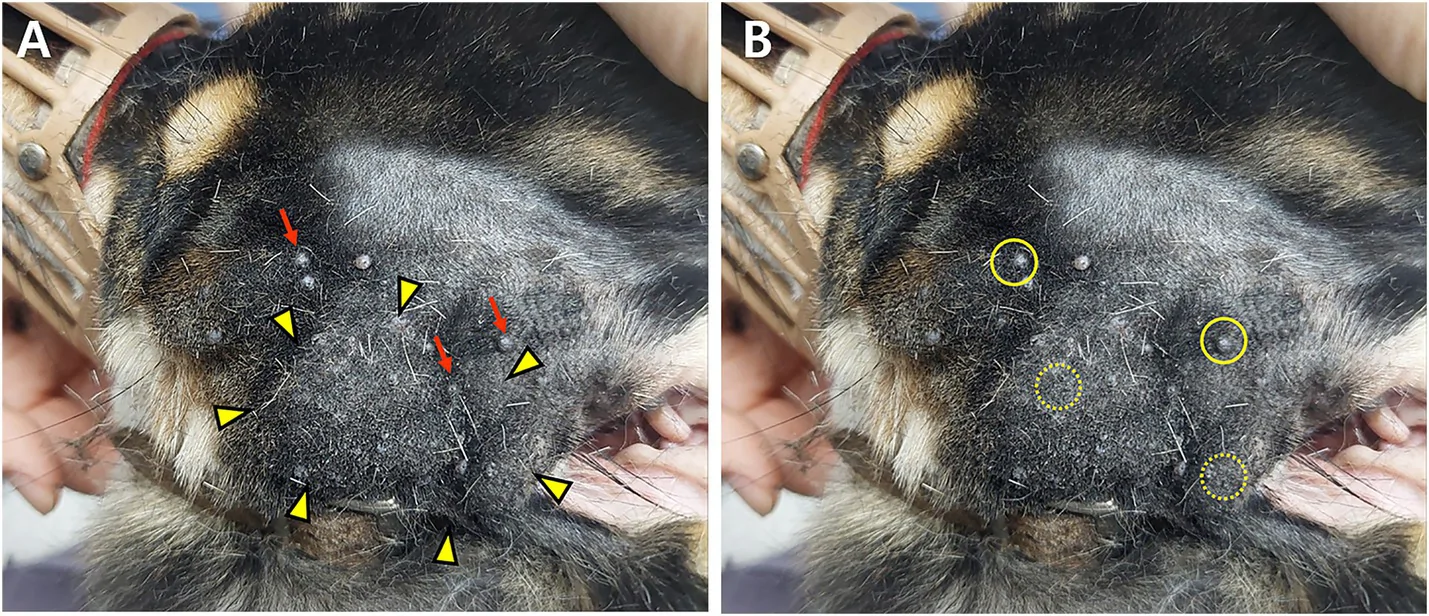
Clinical appearance and biopsy sampling sites of the left facial lesion **(A)** Clinical appearance of the lesion at initial presentation. A firm, black-pigmented, cutaneous nodule (yellow arrow head) measuring approximately 4.0 × 3.8 cm was presented in the left facial region and was well-circumscribed on palpation. Multiple papules (red arrow) were located adjacent to the nodule. **(B)** Punch biopsy sampling sites. Biopsies were obtained from two areas of the nodule (yellow dashed circle) and from two adjacent areas containing papules (yellow solid circle).

For more accurate visualization and measurement, the hair in the left facial region was clipped from the periocular region to the auricular tragus, encompassing the zygomatic arch and the left frontal region. Physical examination revealed a firm, well-circumscribed, raised, pigmented nodule measuring approximately 4.0 × 3.8 cm, located midway between the lateral canthus of the left eye and the left auricular tragus (Figure 1A). Multiple pigmented papules were observed on the surface of the nodule and within the periocular area, specifically between the lateral canthus of the left eye and the nodule. The nodule was firm and non-painful on palpation, with no evidence of ulceration, crusting, discharge, or pruritus. Apart from the cutaneous lesions, no other abnormalities were identified on physical examination.

### 2.2 Diagnostic imaging and staging

**Figure 2:**
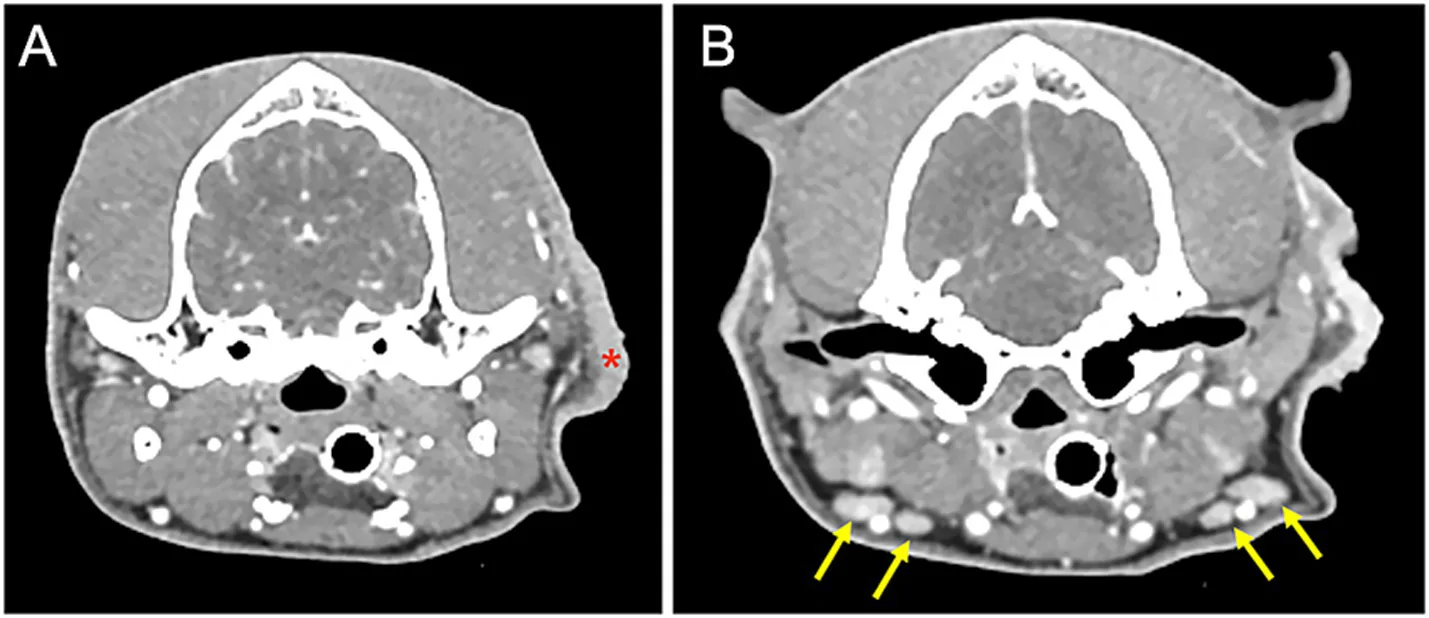
Computed tomographic images of the facial lesion and regional lymph nodes. **(A)** Computed tomography (CT) image showing focal cutaneous thickening (*) up to 5.0 mm at the level of the left ear tragus. The lesion exhibited isoattenuating enhancement relative to the surrounding cutaneous tissue, with attenuation values measured in Hounsfield units (HU) (pre-contrast: 67 HU; arterial: 124 HU; portal: 197 HU; delayed: 215 HU). **(B)** Mild enlargement of regional lymph nodes, including bilateral mandibular (yellow arrow, right: 4.1 mm, left: 4.7 mm), retropharyngeal (right: 4.0 mm, left: 4.6 mm), and superficial cervical nodes (right: 7.7 mm, left: 7.5 mm), was observed on CT.

Lateral skull radiographs were obtained to evaluate for bony involvement before advanced imaging. These showed a partially ill-defined, convex-shaped, soft tissue opacity (3.2 × 1.4 cm) adjacent to the left zygomatic arch at the orbital level. Computed tomography (CT) of the skull, thorax, and abdomen was performed to assess lesion extent and evaluate for regional or distant metastasis. On skull CT, localized cutaneous thickening was identified in the left facial region, centered at the level of the left auricular tragus. The lesion extended axially from the angular process of the mandible to the zygomatic arch and coronally from the zygomatic process of the maxilla to the cranial aspect of the ear canal (Figure 2A). Following intravenous contrast administration, the lesion exhibited isoattenuating enhancement relative to surrounding cutaneous tissue. There was no definitive invasion into the adjacent subcutaneous tissue, temporal or masseter muscles, or the zygomatic bone. Mild enlargement of the regional lymph nodes, including bilateral mandibular, retropharyngeal, and superficial cervical nodes, was observed on CT (Figure 2B). Because the enlargement was mild on CT and the lymph nodes were not clearly palpable, image-guided fine-needle aspiration was not pursued at that time. Diagnostic imaging revealed no pulmonary or visceral metastases. Routine hematology, serum biochemistry, and urinalysis showed no additional abnormalities.

### 2.3 Cytologic, histopathological and immunohistochemical findings

**Figure 3:**
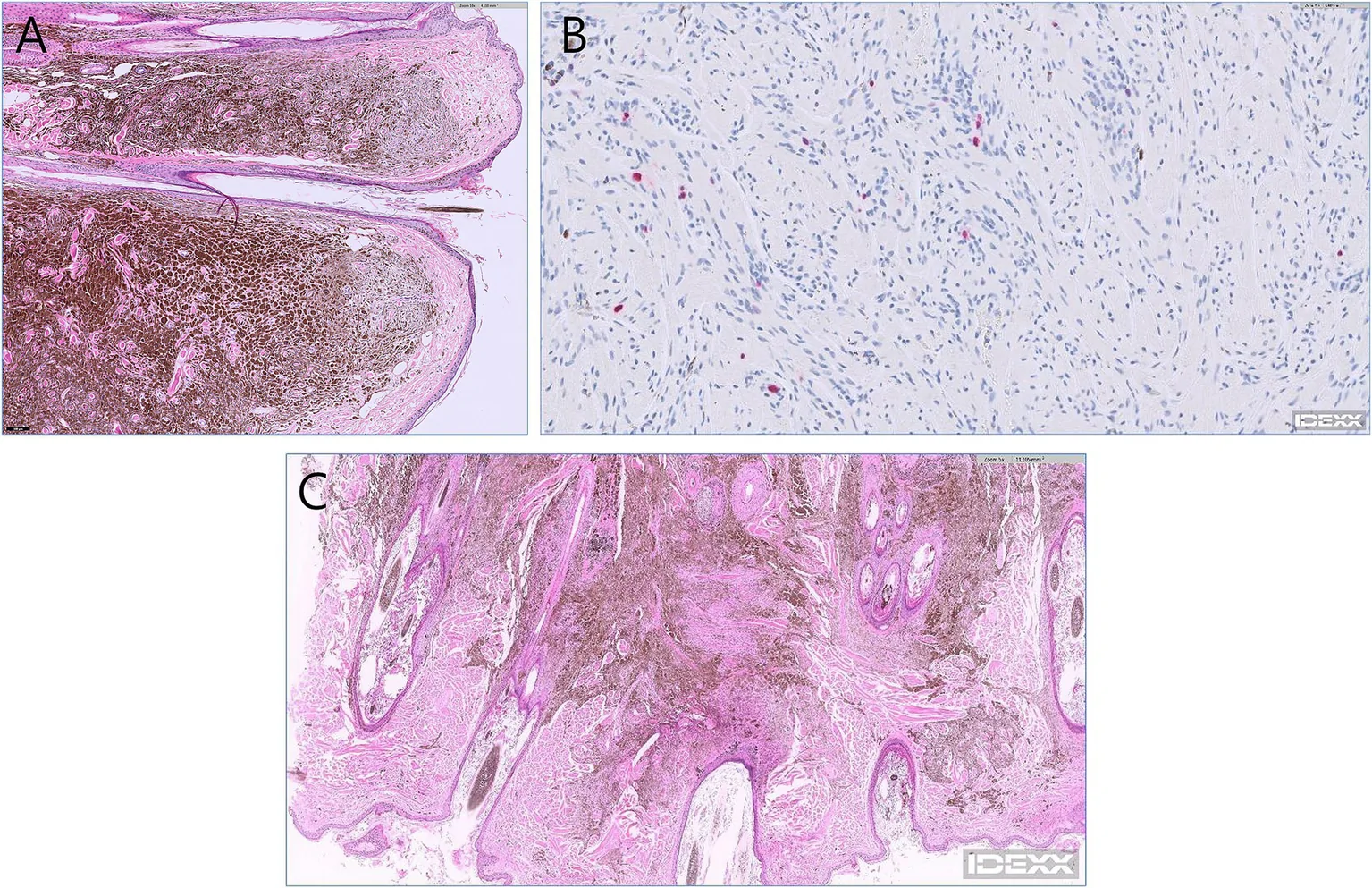
Histopathology and Ki-67 immunohistochemistry of the lesion. **(A)** Histopathology of the left facial nodule. Neoplastic cells were predominantly located within the dermis, with mild extension into the superficial subcutis. Hematoxylin and eosin stain, 10× magnification. **(B)** Ki-67 immunohistochemistry of the biopsy sample. Brown nuclear stain highlights Ki-67 positive tumor cells. Limited nuclear labeling was observed in the neoplastic cells (Ki-67 labeling index <15%). **(C)** Histopathology of the left facial nodule after the 3-month imiquimod withdrawal period. Neoplastic cells remained predominantly located within the dermis, with possible extension into the superficial subcutis, and showed histopathological features similar to those observed in the initial biopsy. Hematoxylin and eosin stain, 5× magnification.

Punch biopsies (4 mm) were obtained from four sites of the lesion for histopathological and immunohistochemical evaluation. Two from the pigmented nodule without papules and two from adjacent papular areas (Figure 1B). Imprint cytology revealed exfoliated cells containing abundant black intracytoplasmic granules, findings suggestive of a melanocytic neoplasm. Histopathology of all four punch biopsies demonstrated similar features of cutaneous melanocytoma, characterized by densely-packed streams and packets of heavily-pigmented neoplastic melanocytes (Figure 3A). Neoplastic cells were predominantly distributed within the dermis, with infiltrative involvement into the superficial subcutis observed in two nodule samples. The subcutis was only partially represented in the punch biopsy samples. Mitotic figures were not observed, nuclear atypia was minimal (<20%), and the Ki-67 index was 5–9% (Figure 3B).

### 2.4 Treatment and follow-up outcomes

Although surgical excision was initially considered, the owner declined further surgery under general anesthesia and preferred a less invasive treatment option. Topical immunotherapy with 5% imiquimod cream (Aldara®; 3M Pharmaceuticals, St Paul, MN, USA) was therefore selected. The cream was applied three times a week at a dose of 250 mg (one single-use sachet) per application, evenly distributed across the clipped lesion area. As no standardized treatment duration has been established for canine melanocytoma, treatment was initially planned for up to 16 weeks based on the reported duration of 4–16 weeks in humans (14). However, the treatment duration was extended to 5 months owing to the owner’s circumstances. The affected lesion was maintained in a clipped state throughout the treatment, and an Elizabethan collar was used to prevent self-trauma and premature removal of the cream.

**Figure 4:**
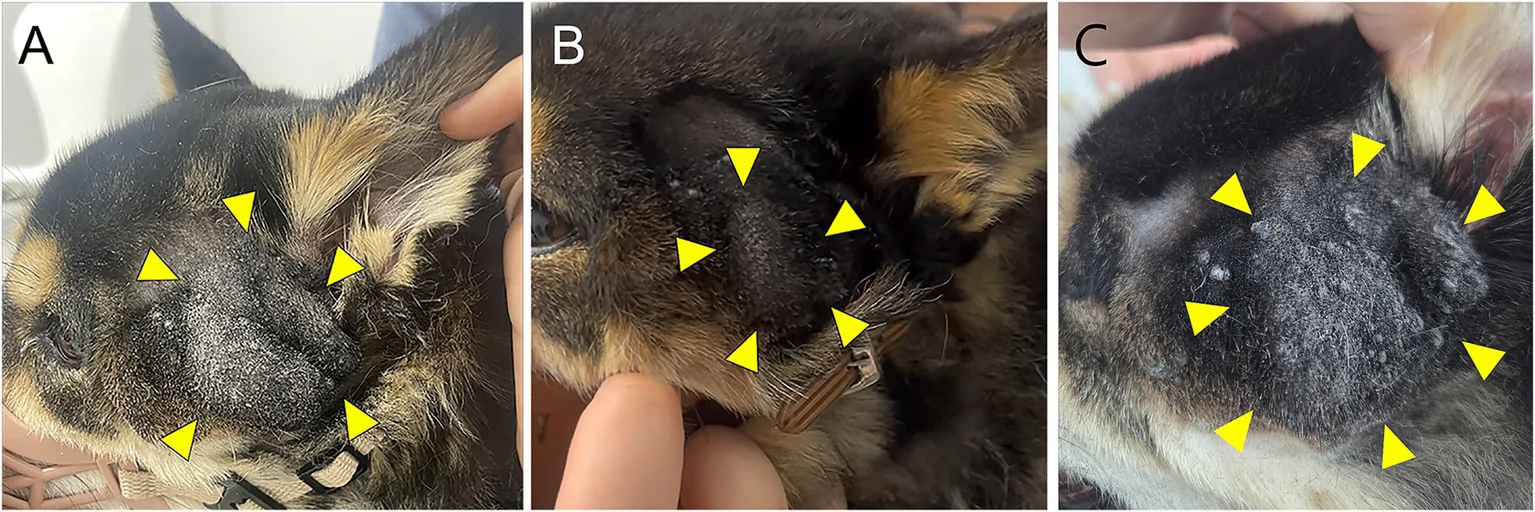
Clinical changes after topical imiquimod treatment **(A)** After 9 weeks of treatment: The left facial nodule (yellow arrowhead) decreased in size to 4 × 3 cm **(B)** After 5 months of treatment. The left facial nodule (yellow arrowhead) decreased in size to 3.3 × 3 cm **(C)** After 3 months treatment withdrawal period, the left facial nodule (yellow arrowhead) had increased in size to 5.0 × 3.6 cm.

The lesions were re-evaluated monthly using calipers by the same clinician, and a progressive reduction in size was observed on serial monthly rechecks. After 1 month of treatment with imiquimod, the nodule decreased in size to 4.0 × 3.5 cm, and the number of black-to-pink papules reduced. At 9 weeks, the nodule measured 4 × 3 cm, and was subjectively softer on palpation compared to previous examinations (Figure 4A). At the end 5 months of the treatment, the nodule decreased in size to 3.3 × 3.0 cm, became softer on palpation, and the number of papules decreased (Figure 4B). No adverse effects of imiquimod, such as erythema, edema, pruritus, or pain, were observed. Regional lymph node palpation was also performed at each follow-up visit, and no palpable enlargement was detected throughout the follow-up period.

A follow-up biopsy was planned after treatment completion, and imiquimod was discontinued for 3 months before biopsy to minimize potential histopathological confounding by treatment-associated microscopic inflammation (9). During this period, the nodule increased in size to 5.0 × 3.6 cm, and the number of papules increased (Figure 4C). Repeat CT performed at the time of the follow-up biopsy showed that the previously enlarged regional lymph nodes were unchanged in size, with no evidence of new pulmonary or visceral metastasis. Two additional 6-mm punch biopsies were obtained, one from the nodule and one from an adjacent papular area. Histopathological findings remained consistent with the initial diagnosis (Figure 3C). The neoplastic cells showed possible subcutaneous extension, with a mitotic count of zero, nuclear atypia in less than 20% of cells, and no lymphovascular invasion. The total follow-up period was 8 months, comprising 5 months of treatment and 3 months after discontinuation of therapy. The owner considered the non-invasive nature of topical imiquimod acceptable and elected to resume treatment after observing lesion enlargement during the withdrawal period. The dog is currently receiving repeated topical imiquimod application, and no adverse effects have been observed.

## 3 Discussion

This case highlights the potential use of topical imiquimod as a palliative or maintenance option for canine cutaneous melanocytoma when complete surgical excision is declined or cannot readily be pursued. The primary treatment for canine melanocytic tumors is surgical excision. For malignant melanocytic tumors, adjuvant therapy, such as radiotherapy, chemotherapy, and immunotherapy, may be considered in patients at high risk of developing microscopic disease and subsequent distant metastasis (3, 5). However, even benign melanocytic tumors may require alternative approaches when surgical removal is limited by anatomical constraints (15).

Imiquimod is a topical immunotherapy that acts as a TLR 7/8 agonist, activating signaling pathways that induce immune modulation and exert direct pro-apoptotic effects on tumor cells (7, 16, 17). A previous study reported that topical 5% imiquimod cream applied three times a week for 3–4 week produced partial clinical and histological regression with long-term maintenance in canine cutaneous melanocytoma (15). In this case, the broad region of the left face made complete surgical excision challenging. Although reconstructive techniques, including local flaps or skin grafts, or alternative modalities such as electrochemotherapy or partial surgical excision were considered, they were declined by the owner due to the need for general anesthesia or the likelihood of incomplete margins. Therefore, topical imiquimod was selected as a non-invasive palliative approach. Its principal advantage in this setting was the ability to provide active local management without additional general anesthesia, extensive tissue excision, reconstructive surgery, or postoperative wound care. Thus, although it was not considered superior to surgical excision, topical imiquimod broadened the available treatment options when definitive surgery was not acceptable to the owner.

During the 5 months treatment period, the facial nodule decreased in size, became subjectively softer on palpation, and the number of surrounding papules decreased. However, these findings should be interpreted as temporary clinical improvement rather than definitive tumor regression. Follow-up biopsy was performed to evaluate histopathologic decrease in neoplastic cell infiltration or other microenvironmental alterations induced by imiquimod therapy. To reduce the possibility that treatment-associated microscopic inflammation could interfere with histopathological interpretation, the post-treatment biopsy was performed 3 months after imiquimod cessation (9). During this period, the facial lesion increased in size and became subjectively firmer, indicating regrowth of the lesion. Histopathology confirmed cutaneous melanocytoma, with findings unchanged from the initial biopsy. The mild extension into the subcutis, previously identified as a negative prognostic indicator (2), was also consistently observed. The Ki-67 index was not re-assessed; however, the persistent subcutaneous extension of neoplastic cells indicated that the follow-up biopsy retained histopathological features similar to those observed initially. No inflammatory infiltrates were observed, reducing concern that treatment-associated inflammation substantially affected histopathological interpretation.

Histopathological examination, with or without immunohistochemistry, is essential for definitive diagnosis and serves as a key prognostic indicator in canine cutaneous melanocytic tumors, encompassing both benign melanocytomas and malignant melanomas (5). A poor prognosis is typically defined by a mitotic index of ≥3/10 high-power fields (hpf), nuclear atypia in ≥20% of cells, and a Ki-67 index of ≥15%. Infiltration limited to the dermis is favorable, whereas extension into the subcutis often indicates more aggressive behavior. Pigmentation involving threshold of ≥50% pigmented cells has been associated with longer survival times and a more favorable prognosis (2, 5, 18). In this case, histopathological findings were consistent with cutaneous melanocytoma, including a mitotic count of zero, nuclear atypia in less than 20% of the cells, the absence of lymphatic invasion, and the presence of highly pigmented melanocytes. The Ki-67 index was less than 15%. Gross prognostic factors included absence of ulceration and lesion thickness was less than 7.5 mm (2, 5, 18). However, despite these benign indices, focal extension into the superficial subcutis was identified. Although biopsy depth limited full assessment of infiltration, and recurrence of the lesion distinguishes it from typical cutaneous melanocytomas.

The clinical course in this case differed from a previous report of topical imiquimod in canine cutaneous melanocytoma. While the previous report described clinical resolution without recurrence after treatment discontinuation (15), our case showed regrowth following treatment cessation after only partial clinical regression. This discrepancy may be due to the unique features of this case, including broad facial involvement, surrounding papular lesions, recurrent presentation, and histological evidence of subcutaneous extension. Therefore, the response in this case is best interpreted as temporary clinical control rather than curative treatment. Clinically, this case suggests that topical imiquimod was associated with a measurable reduction in lesion size; however, enlargement after treatment discontinuation indicates that its effect may be temporary and that maintenance therapy or close monitoring may be required for long-term clinical control.

This case has several limitations. Although mild enlargement of the bilateral mandibular, retropharyngeal, and superficial cervical lymph nodes was identified on initial CT, reactive lymphadenopathy was considered more likely than metastatic involvement based on the mild degree and bilateral distribution of the enlargement. Nevertheless, given the focal extension of the tumor into the superficial subcutis, lymph node sampling would have allowed a more complete assessment. As this study is based on a single patient, the response to imiquimod may not be representative of all canine cutaneous melanocytic tumors. Furthermore, treatment evaluation was limited to subjective visual inspection, palpation, and size measurements rather than advanced imaging or standardized assessment tools. Nevertheless, distinct changes in the lesion were observed during both treatment and cessation. Additional cases with complete clinical staging and longer follow-up are required to determine whether topical imiquimod can provide sustained clinical control in canine cutaneous melanocytic tumors.

## 4 Conclusion

This case suggests that topical imiquimod may provide temporary clinical improvement in canine cutaneous melanocytoma when surgical excision is not feasible. The reduction in lesion size during treatment, followed by enlargement after discontinuation, suggests that imiquimod should be considered as a palliative or maintenance option rather than a curative therapy. Careful histopathological evaluation, assessment of prognostic indicators, and long-term follow-up remain important, particularly in recurrent lesions or those showing superficial subcutaneous involvement.

## Data Availability

The raw data supporting the conclusions of this article will be made available by the authors, without undue reservation.
